# Supplementary material for: Infectious potential and circulation of SARS-CoV-2 in wild rats
Source: PLoS One. 2025 May 12;20(5):e0316882. doi: 10.1371/journal.pone.0316882 (PMC12068656; doi:10.1371/journal.pone.0316882)
Supplement: S1 File — (DOCX) [file pone.0316882.s001.docx]

**S1_File. ACE2 alignments of *Homo sapiens*, *Rattus norvegicus* and *Rattus rattus*.**

Only diverging amino acids are representing.

Consensus MSSSXWLLLSLVAVATAQSLIEEKAESFLNKFNQEAEDLSYQSSLASWNYNTNITEENAQ 60

ACE2 Homo sapiens ....S.........TA...T...Q.KT..D...H.....F..................V. 60

ACE2 Rattus rattus ..R.P....................................................... 60

ACE2 Rattus norvegicus ....C....................................................... 60

Consensus KMNEAAAKWSAFYEEQSKIAQNFSLQEIQNATIKRQLKALQQSGSSALSPDKNKQLNTIL 120

ACE2 Homo sapiens N..N.GD.....LK...TL..MYP......L.V.L..Q....N...V..E..S.R..... 120

ACE2 Rattus rattus .............................D.............................. 120

ACE2 Rattus norvegicus ............................................................ 120

Consensus NTMSTIYSTGKVCNSMNPQECFLLEPGLDEIMATSTDYNRRLWAWEGWRAEVGKQLRPLY 180

ACE2 Homo sapiens ..............PD.....L......N....N.L...E......S..S.......... 180

ACE2 Rattus rattus ......................V..................................... 180

ACE2 Rattus norvegicus ............................................................ 180

Consensus EEYVVLKNEMARANNYEDYGDYWRGDYEAEGVEGYNYNRNQLIEDVENTFKEIKPLYEQL 240

ACE2 Homo sapiens ..............H.............VN..D..D.S.G.......H..E.......H. 240

ACE2 Rattus rattus ............................................................ 240

ACE2 Rattus norvegicus ............................................................ 240

Consensus HAYVRTKLMXVYPSYISPTGCLPAHLLGDMWGRFWTNLYPLTTPFLQKPNIDVTDAMVNQ 300

ACE2 Homo sapiens .....A...NA.......I....................S..V..G............D. 300

ACE2 Rattus rattus .........D.................................................. 300

ACE2 Rattus norvegicus .........E.................................................. 300

Consensus SWDAERIFKEAEKFFVSVGLPQMTPGFWTNSMLTEPGDDRKVVCHPTAWDLGHGDFRIKM 360

ACE2 Homo sapiens A...Q................N..Q...E.....D..NVQ.A..........K.....L. 360

ACE2 Rattus rattus ............................................................ 360

ACE2 Rattus norvegicus ............................................................ 360

Consensus CTKVTMDNFLTAHHEMGHIQYDMAYAKQPFLLRNGANEGFHEAVGEIMSLSAATPKHLKS 420

ACE2 Homo sapiens .......D..................A................................. 420

ACE2 Rattus rattus ............................................................ 420

ACE2 Rattus norvegicus ............................................................ 420

Consensus IGLLPSNFQEDNETEINFLLKQALTIVGTLPFTYMLEKWRWMVFQDKIPREQWTKKWWEM 480

ACE2 Homo sapiens ....SPD.....................................KGE..KD..M...... 480

ACE2 Rattus rattus ...........D............I.............................Q..... 480

ACE2 Rattus norvegicus ............................................................ 480

Consensus KREIVGVVEPLPHDETYCDPASLFHVSNDYSFIRYYTRTIYQFQFQEALCQAAKHDGPLH 540

ACE2 Homo sapiens ..........V............................L...............E.... 540

ACE2 Rattus rattus ............................................................ 540

ACE2 Rattus norvegicus ............................................................ 540

Consensus KCDISNSTEAGQKLLNMLSLGNSGPWTLALENVVGSRNMDVKPLLNYFQPLFVWLKEQNX 600

ACE2 Homo sapiens ..............F...R..K.E...........AK..N.R......E...T...D..K 600

ACE2 Rattus rattus ...........................................................S 600

ACE2 Rattus norvegicus ...........................................................R 600

Consensus

NSTVGWSTDWSP--YADQSIKVRISLKSALGXXAYEWTDNEMYLFRSSVAYAMREYFSRE 658

ACE2 Homo sapiens

..F.........--.................DK....N................Q..LKV 658

ACE2 Rattus rattus

......N.....CDFT.CT--------------H.......................... 646

ACE2 Rattus norvegicus

............--.................KN........................... 658

Consensus KNQTVPFGEADVWVSDLKPRVSFNFFVTSPKNVSDIIPRSEVEEAIRMSRGRINDIFGLN 718

ACE2 Homo sapiens ...MIL...E..R.AN....I.......A..........T...K......S....A.R.. 718

ACE2 Rattus rattus ............................................................ 706

ACE2 Rattus norvegicus ............................................................ 718

Consensus DNSLEFLGIYPTLKPPYEPPVTIWLIIFGVVMGXVVVGIVILIVTGIKGRKKKNETKREE 778

ACE2 Homo sapiens .........Q...G..NQ...S....V......VI........F...RD.....KARSG. 778

ACE2 Rattus rattus .................................M.......................... 766

ACE2 Rattus norvegicus .................................T.......................... 778

Consensus

NPYDSXDIGKGESNAGFQNSDDAQTSF 805

ACE2 Homo sapiens

...A.I..S...N.P....T..V.... 805

ACE2 Rattus rattus

.....V..................... 793

ACE2 Rattus norvegicus

.....M..................... 805
