# Supplementary material for: Infectious potential and circulation of SARS-CoV-2 in wild rats
Source: PLoS One. 2025 May 12;20(5):e0316882. doi: 10.1371/journal.pone.0316882 (PMC12068656; doi:10.1371/journal.pone.0316882)
Supplement: S2 File — (DOCX) [file pone.0316882.s002.docx]

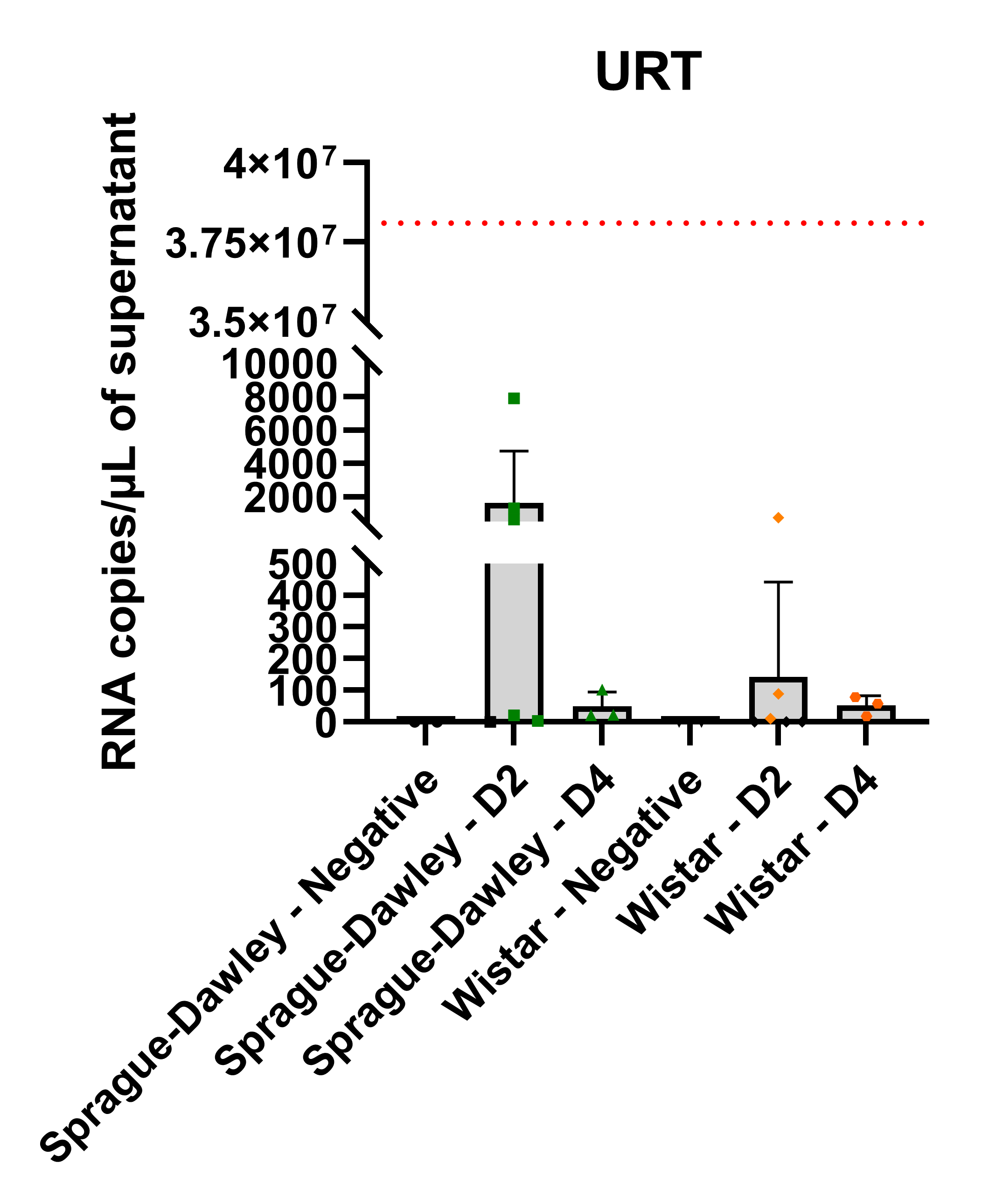


**S2_File. Viral RNA load in the upper respiratory tract (URT) of the Sprague-Dawley and Wistar groups at 2 and 4 days post-inoculation.**

Positive rats are shown in color, green for Sprague-Dawley and orange for Wistar. The number of RNA copies of the BA.5 strain inoculated into the rats is represented as red dots. The means are represented ± one standard deviation.
